# Undergraduate students’ contributions to health service delivery through community-based education: A qualitative study by the MESAU Consortium in Uganda

**DOI:** 10.1186/s12909-016-0626-0

**Published:** 2016-04-25

**Authors:** Lynn M. Atuyambe, Rhona K. Baingana, Simon P. S. Kibira, Anne Katahoire, Elialilia Okello, David K. Mafigiri, Florence Ayebare, Henry Oboke, Christine Acio, Kintu Muggaga, Scovia Mbalinda, Ruth Nabaggala, Gad Ruzaaza, Wilfred Arubaku, Samantha Mary, Peter Akera, James K. Tumwine, David H. Peters, Nelson K. Sewankambo

**Affiliations:** Department of Community Health and Behavioural Sciences, Makerere University School of Public Health, College of Health Sciences, New Mulago Hospital Complex-School of Public Health Building Suite nr 307, P.O. Box 7072, Kampala, Uganda; School of Biomedical Sciences, Makerere University College of Health Sciences, Kampala, Uganda; School of Medicine, Makerere University College of Health Sciences, Kampala, Uganda; School of Social Sciences, Makerere University College of Humanities and Social Sciences, Kampala, Uganda; Faculty of Medicine, Gulu University, Kampala, Uganda; Faculty of Medicine, Mbarara University of Science and Technology, Mbarara, Uganda; School of Health Sciences, Kampala International University Western Campus, Bushenyi, Uganda; Office of the Principal, Makerere University College of Health Sciences, Kampala, Uganda; Department of International Health, Johns Hopkins Bloomberg School of Public Health, Baltimore, USA

**Keywords:** Undergraduate students, Contribution, Health service, Community-based education, Qualitative, MESAU, Uganda

## Abstract

**Background:**

It has been realised that there is need to have medical training closer to communities where the majority of the population lives in order to orient the trainees’ attitudes towards future practice in such communities. Although community based education (CBE) has increasingly been integrated into health professions curricula since the 1990s, the contribution students make to service delivery during CBE remains largely undocumented. In this study, we examined undergraduate health professions students’ contribution to primary health care during their CBE placements.

**Methods:**

This was a qualitative study involving the Medical Education for Equitable Services to All Ugandans consortium (MESAU). Overall, we conducted 36 Focus Group Discussions (FGDs): one each with youth, men and women at each of 12 CBE sites. Additionally, we interviewed 64 community key-informants. All data were audio-recorded, transcribed and analysed using qualitative data analysis software *Atlas.ti Ver7*.

**Results:**

Two themes emerged: students’ contribution at health facility level and students’ contribution at community level. Under theme one, we established that students were not only learning; they also contributed to delivery of health services at the facilities. Their contribution was highly appreciated especially by community members. Students were described as caring and compassionate, available on time and anytime, and as participating in patient care. They were willing to share their knowledge and skills, and stimulated discussion on work ethics. Under the second theme, students were reported to have participated in water, sanitation, and hygiene education in the community. Students contributed to maintenance of safe water sources, educated communities on drinking safe water and on good sanitation practices (hand washing and proper waste disposal). Hygiene promotion was done at household level (food hygiene, hand washing, cleanliness) and to the public. Public health education was extended to institutions. School pupils were sensitised on various health-related issues including sexuality and sexual health.

**Conclusion:**

Health professions students at the MESAU institutions contribute meaningfully to primary health care delivery. We recommend CBE to all health training programs in sub-Saharan Africa.

## Background

Community-based education (CBE) involves the integration of education and practice in the community within the learning process [1]. CBE arose from the increasing need to address the universal right to health and contributes to the “Health for All” strategy by promoting primary health care (PHC) [2]. The aim of CBE is to expand students’ insight of community health problems through their learning, service and research in the community and thereby improve community health [3]. Three of the principles of CBE are key to this study: (i) CBE is a standard, integral, and continuing part of the educational process, program and curriculum, not a peripheral or casual experience; (ii) the community must be actively involved in the educational program and there must be clear benefits to both the student and the community; (iii) the students’ work during training must be “real work” that is related to their educational needs, and also forms part of the requirements for qualification [1]. CBE is part of the larger concept of competency-based education which emphasizes outcome-based instruction that is adaptive to the changing needs of students, teachers, and the community. The competencies should describe the student’s ability to apply basic and other skills in situations that are commonly encountered in everyday health professional practice.

It is well recognised that students in clinical settings in teaching hospitals contribute to service delivery and quality of services [4, 5]. As the students contribute their much appreciated services in the clinics, they in return gain practical knowledge and skills [6, 7] making their participation mutually beneficial. In sub-Saharan Africa and other low income settings, this contribution is in the context of limited human resources for health especially at peripheral health facilities. CBE has increasingly been integrated into health professions curricula since the 1990s, and it is evident from studies in Indonesia [8], Nigeria [9, 10] and Uganda [11] that community members appreciate CBE and deem the students’ activities as beneficial. Community members report that they have seen improvements in health and health seeking behaviours and increased community participation in PHC [10, 11]. While the students’ contribution at the teaching hospitals is well documented, there is a dearth of literature regarding how students may specifically be contributing to service delivery especially PHC during CBE. We undertook this study to document the students’ contribution to PHC through community-based education.

## Methods

### Study design

This was a qualitative study in which Key Informant Interviews (KIIs) and Focus Group Discussions (FGDs) were conducted.

### Setting

Uganda currently has 6 pre-service medical training institutions. In 2010, five of them: Makerere University College of Health Sciences (MakCHS), Gulu University (GU), Mbarara University of Science and Technology (MUST), Kampala International University (KIU) and Busitema University (BU) came together to form Medical Education for Equitable Services to All Ugandans consortium (MESAU) with funding from the US Government-supported Medical Education Partnership Initiative (MEPI) and technical support from Johns Hopkins University [12, 13]. MESAU is the first nation-wide consortium approach to addressing medical education in Uganda with the overall aim of standardising medical education and developing the partner institutions as centres of excellence for medical education, research and service that address local and national needs to improve health in Uganda. One of MESAU’s objectives is to improve the quality and relevance of medical education in order to produce health workers with the competencies and motivation to deliver locally relevant services. Each of the MESAU institutions has implemented CBE as an integral part of their respective curricula for varying lengths of time since 1989. Community-based education, research and service (COBERS), the MESAU model of CBE, is a key performance area for the consortium institutions. Although the MESAU institutions place their students for community exposure in different years of study [14], they have common goals and site selection criteria for COBERS. Before students go to the sites, they are briefed and are given overview lectures that introduce them to community health, PHC and what to expect during their COBERS attachment.

### Study population and sites

The study populations included health workers and community members. In 2011, each institution selected new COBERS placement sites for the larger COBERS impact evaluation study. Gulu University selected 5 new sites, KIU and MUST selected 10 new sites each, while MakCHS selected 20 new sites. These sites were used for the first time for student COBERS placements before collection of data for this study was carried out. Twelve of these sites were purposively selected from different districts and regions for this study (Table 1). These sites were used as entry points to select respondents from the communities within a five kilometre radius from the health facility.

**Table 1:** COBERS placement sites involved in the study

Institution	Health Facility	District	Region
Gulu University	Koch Goma HCIII	Amuru	North
	Agoro HCIII	Lamwo	North
Kampala International University	Bitooma HCIII	Bushenyi	South west
	BiterekoHCIII	Mitooma	South west
Mbarara University of Science and Technology	Kazo HCIII	Mbarara	South west
	Rubanda HCIII	Kabale	South west
Makerere University College of Health Sciences	Nebbi Hospital	Nebbi	West Nile
	Amai HCIV	Amolatar	North
	Nsinze HCIV	Namutumba	East
	Namungalwe HCIV	Iganga	East
	Nyenga Hospital	Buikwe	Central
	Mpigi HCIV	Mpigi	Central

Key: *HC* Health Centre

### Methods of data collection

Data were collected using KIIs and FGDs (Table 2) with guides developed by a team of faculty from the MESAU institutions led by those with qualitative research expertise. The key questions and discussion points for the KIIs and FGDs were the students’ activities at the health facilities and in the communities during COBERS, their contributions to community health, and whether they affected functioning at the health facilities.

**Table 2:** Data collected

	*No. of sites selected*	Community FGDs		Community OPL KIIs		Health Facility KII	
		*Planned*	*Achieved*	*Planned*	*Achieved*	*Planned*	*Achieved*
Gulu	2	6	6	6	6	6	6
KIU	2	6	6	6	5	6	5
MakCHS	2	18	18	18	14	18	18
MUST	6	6	6	6	5	6	5
Total	12	36	36	36	30	36	34

Key: *FGD* Focus Group Discussions, *OPL* Opinion Leaders, *KII* Key Informant Interviews

#### Key informant interviews

Overall, we conducted 64 KIIs in the community. Specifically, we conducted 30 interviews with opinion leaders such as the Local Council I Chairpersons and members of Village Health Teams (VHT). These opinion leaders were purposively selected from communities where students were placed. We also interviewed 34 health facility staff involved in student activities at the selected COBERS sites.

#### Focus group discussions

We conducted 36 FGDs: one each with youth, men and women at each of the 12 selected sites. Focus groups participants were purposively selected from communities that receive students during community placement.

### Training and quality control

Experienced research assistants were recruited and re-trained on qualitative data collection methods and tools. They worked in pairs: one moderated the FGD or interviewed the key informant while the other took notes. All discussions and interviews were audio recorded. Each session lasted about an hour. Participants were provided with a soft drink and a snack and did not receive any monetary compensation. Data was collected between August 2012 and January 2013.

### Data management and analysis

All interviews/discussions were transcribed verbatim and the transcripts were imported into *Atlas.ti 7* [15]. A team approach was used for data analysis. The team developed an analysis plan based on the objectives of the study and generated a joint coding scheme using this analysis plan after reading a sample of the transcripts. Code definitions were agreed on to minimise bias and enhance coding consistency. A team project was created in *Atlas.ti* and later split for coding by teams from each institution. We used the coding scheme we developed but allowed open coding for emerging codes which we agreed on and included. The codes were independently examined by an external reviewer who was not part of the team that generated them initially. After coding, the hermeneutic units were merged again for analysis. We run query reports for each theme and used them in writing the results. Primary documents matrices were also produced to help understand patterns in the data across the four MESAU institutions’ transcripts.

### Ethical considerations

The study was approved by MakCHS School of Medicine Ethics Review Committee and was registered with Uganda National Council for Science and Technology (Registration SS 2748). The study rationale, objectives, potential risks and benefits and participant rights to withdraw from the study anytime without affecting services access were explained to participants. All study participants provided written informed consent.

## Results

**Fig. 1:**
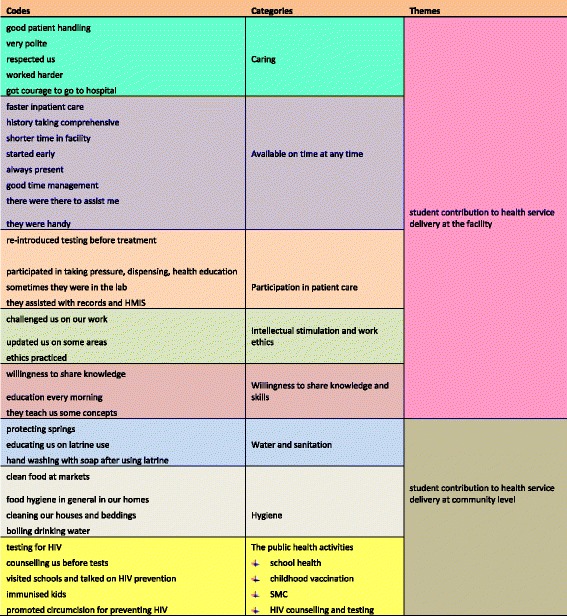
An exemplar of the analytical framework

Two themes, namely, students’ contribution at health facility level and students’ contribution at community level emerged from the data. Our results show that students were not only learning; they were also contributing to health service delivery. Their contribution was highly appreciated especially by community members. We also established that the presence of students at the facilities had both positive and negative effects on their functioning (Fig. 1).

### Students’ contribution to health service delivery at the facilities

Students’ contribution at the health facilities was described in various ways which we grouped into five categories during analysis. Students were described as: being caring and compassionate, available on time and anytime, participating in patient care, willing to help and share their knowledge and skills, and stimulating discussion on various topics in health as well as inspiring health workers regarding work ethics.

#### Caring and compassionate

‘A caring attitude’ was commonly used to describe students’ behaviour at the health facilities. Community members reported that they were warmly received by the students, who made them feel welcome and handled them with kindness and respect as illustrated by the following quotes:

*“How they handled patients was exemplary I think their greatest contribution here was showing the community that they were wanted and that they are also human beings. If all medical workers worked that way, patients would even get well without treatment”* FGD, Men, GU

*“They did a wonderful job… they talk politely even if others do not know the language not like these usual Doctors and Nurses who talk in a rude way and sometimes you may not go back again to get treatment for fear of rudeness.”* FGD, Women, MUST

Students were admired for their ‘professionalism’ which the community and facility staff said was displayed through their verbal and non-verbal communication and respect for patients. The community noted that this attitude influenced the regular health facility staff who became more committed, worked harder and managed their time better.

*“It also brought a big change in the work and attitudes of both the health workers who became more committed and worked harder hence people were served so quickly and also the community attitudes changed and they started coming to the health unit much more given the change in the quality of service around”*. KII, Health Facility Staff, GU

As a result, patients were served better and faster, which in turn led to the communities having a better attitude towards the facilities. The positive relationship between the facilities and the public served to “pull” community members to the facilities, while the students’ community activities “pushed” community members to the facilities. Consequently, public demand for services at health facilities increased as illustrated by the quotes below:

*“We used to fear going to hospital, but from the time we found the students there we got courage to go to hospital. We frequent the hospital to get treatment. Otherwise a lot of change was brought by students.”* FGD, Men, MakCHS

*“When these students are around, they encourage the community members to visit the health centre. Some people actually visit the health centre because of those students and when they leave, such people also stop going for treatment”* KII, Opinion Leader, MUST

#### Available on time and at any time

Students, according to the community members, were available not only on time, but anytime. There were reports about reduction in waiting time at the health facilities as well as improved access to services due to flexible working hours. Furthermore, time management was incorporated into the day-to-day practices of some health facilities. This was particularly highlighted by community members as illustrated below:

*“The way of serving people so fast because there were so many workers which made patients history taking, getting drugs to be done very fast. So people did not take long here. As you can see today there are many people but only a few health workers who cannot serve with the same pace if they were many with division of labour”* KII, Opinion Leader, GU

*“They improved on the time we spend in the hospital. They start work very early and they also work fast enough to enable patients go back home early. Besides they were always present unlike the other health workers”* FGD, Women, MakCHS

*“The most visible change is the improvement on time management by the health facility staff because the students would be at the facility by around 7:30 to 8:00 am which we ultimately also coped with.”* KII, Health Facility Staff, GU

Participants also noted that when students were present, there was always someone available at the health facility to attend to patients.

#### Participation in patient care

Students were described as “extra hands” by the health workers because they participated in various clinical and non-clinical activities. Students re-introduced services such as weighing patients, measuring their blood pressure and body temperature, and some laboratory tests that were not being provided at some facilities due to various constraints:

*“The students also introduced testing for most illness before prescribing treatment when they came to the facility. In the past we could just give medicine without testing as long as the symptoms seem clear to you, but with the coming of the students, all diseases must be tested before any prescription is made”.* KII, Health Facility Staff, GU

Students were involved clinical measurements, specifically pulse rate, temperature, respiration rate, and blood pressure. They also participated in immunization, dispensing drugs and providing some assistance during surgical procedures such as male circumcision.

*“When I visited those at the lab they were participating in getting samples like for malaria and others. And in OPD (out-patient department) some were at the clinic working hand in hand with the clinicians. They would observe how the clinicians prescribe and those in OPD would record. The patients come with their medical forms so they would record in OPD registers and observe how they were dispensing”* KII, Health Facility Staff, MakCHS

*“Sometimes they were dispensing drugs, they were in the laboratory and in the maternity because when tasks were given out and sometimes one is in the pharmacy, sometime he would be taking patient information from OPD and others were in the ward”.* KII, Opinion Leader, GU

Students also assisted with records including filling Health Management Information System (HMIS) forms, producing monthly reports, registration of out-patients and filling antenatal cards (books).

*“They were there to assist me. Giving health education talks. Counselling these mothers. Giving them information. They were doing something. They were helping because if we are few and he is there, he is able to help as an assistant. I can send him give me this, take this patient for scan, take this one to the lab etc. They do a lot of work”.* KII, Health Facility Staff, MakCHS

#### Intellectual stimulation and work ethics improvements

The students’ presence at the health facility stimulated and challenged some health care providers to seek new information in order to provide better services to patients. Their presence also re-energised health workers because of the extra hands as well as the students’ work ethic as noted by this health worker:

*“When you have young men and women from the higher institution, you feel challenged. You feel you need to know more. You feel the need to keep up to date with current information. So it stimulates my search for more information about that particular subject of interest.”* KII, Health Facility Staff, MUST

*“Yes, yes……indeed it has especially when you take on the teaching role, you get challenged to do research, reading sometimes on internet on topics to teach students thereby improving on my practice.”* KII, Health Facility Staff, MakCHS

#### Health education activities

COBERS students were recognized for the health education sessions that they offered both at health facilities and in the communities. Sometimes due to human resource constraints at the health facilities and heavy workload, health education is not conducted. Students filled this gap, providing health education at the health facilities and during outreach and home visits about issues such as family planning, maternal and child health, sanitation and hygiene. This was appreciated by the communities.

*“They gave good health education every morning as the patients waited for services to start or as the patients waited for other staff. I greatly benefitted from such talks especially on malnutrition in pregnant women”* FGD, Women, GU

*“Those students have been teaching us most of the health issues, how we are supposed to live, we should always wash our hands before we eat, after visiting the toilet, we should not have dirty surroundings…”* FGD, Men, MakCHS

#### Willingness to share knowledge and skills

The presence of COBERS students provided health staff the opportunity to obtain (informal) continuing professional development as well as continuous medical education. Students shared their experiences and up-to-date information learnt while at their respective institutions.

“*They were also involved in CMEs (Continuous Medication Education) with us”* KII, Health Facility Staff, MakCHS

*“..they came up with a graph showing the common diseases and sicknesses that affect our people in our communities here and they found out that the most common was malaria, followed by cough and flue and respiratory diseases, acute diarrhea…so that gave me a picture that when I collect data I should also be analysing it something, I learnt from them”* KII, Health Facility Staff, MakCHS

*“Yes at one point, I learnt some prescriptions that I didn’t know from them especially in treating children with tuberculosis. I must say although I was responsible for their learning while in the maternity ward, I also learnt a lot from them”.* KII, Health Facility Staff, GU

### Student contribution to health service delivery at community level

COBERS students participated in various community health activities in the areas of water and sanitation and hygiene. Students contributed to maintenance of safe water sources, educated communities on drinking safe water in the households and on good sanitation practices including latrine construction, hand washing and appropriate waste disposal. Hygiene was promoted at household level and at community level for example among food handlers in markets. Public health education extended to institutions such as schools where sensitisation on various health-related issues including sexuality and sexual health was conducted.

#### Water and sanitation

COBERS students’ activities in the community were perceived to bring about general improvement in sanitation and hygiene as expressed in the quotes below:

*“The doctors* [students] *helped us a lot to keep clean in our homes, they taught us how to build drying racks, to dig rubbish pits and they dug for those who were not strong enough to dig, they told us to dig latrines and keep them clean, to wash hands before eating and us the people to bathe*” FGD, Youth, MakCHS

*“Sanitation in this community was so alarming in that most of the people would not wash their beddings especially for children, bushes were not cleared and worst of all we would wash our hands in the same container not knowing the impact it could cause. But now I am amused about people’s positive response towards these activities”* KII, Opinion Leader, MUST

#### Other public health activities

Students also participated in providing services at community level such as HIV counselling and testing. Health workers and community leaders reported that many people were tested for HIV and were vaccinated against childhood diseases.

*“Their education to the community especially on HIV testing was very good. There is a real increase in the number of people testing for HIV and the number of youths coming for safe male circumcision has also shot up. Actually I now spend more time in the hospital to counsel people than before because of the high number”.* KII Opinion Leader, MakCHS

A noteworthy contribution of the students as reported by key informants at some sites was providing feedback after community activities. This feedback was provided verbally to the community leaders and in writing to the district health team and the health facilities.

*“Apart from health education that they offer to the community, after visiting the community they come back and write reports and give us these reports. So from those reports, they also meet village health team members (VHTs). When they come, they give us those reports in which they make recommendations and we give them to VHTs so that they can go and make a difference and act on them”.* KII, Health Facility Staff, MakCHS

*“Then after rotating through all the homes of people, they get time to gather all the people in the village in a defined place and provide them with feedback”.* KII, Health Facility Staff, MUST

As a result of students’ community health activities, community respondents believed that improvements were registered in a number of areas related to community health such as hygiene, nutrition, awareness and knowledge of general disease prevention, as well as in general health service coverage.

### Challenges faced during field placement

The presence of students at the health facilities was not without some challenges. Some health workers felt that the students presented extra workload because they had to be supervised. Others reported that they spent more time on each patient because they had to explain to students as they provided patient care:

*“When the students were around it was more of coaching them during the ward rounds and this increased time spent on patients. Otherwise you would not have taken that long”* KII, Health Facility Staff, MakCHS

In addition, some key informants noted that some patients did not appreciate being attended to by students with the effect that the number of mothers coming to the facility for delivery during COBERS placement was reduced:

*“Staff members have the burden of convincing the patients to be attended by the student”* KII, Health Facility Staff, KIU.

*“The functioning of the health unit has been affected negatively for example the number of mothers coming for delivery especially has reduced due to the negative attitude they have that students are going to learn from them. When students are here we really get few patients especially in the maternity because the mothers are not comfortable to be attended by the students that they are not yet qualified”* KII, Health Facility Staff, KIU.

While the purpose of COBERS is to provide the opportunity for students to learn under apprenticeship and with the supervision of health workers, there were a few health workers who perceived the presence of COBERS students as an opportunity to take unofficial leave thus leaving students unsupervised.

*“Yes, I also get some time to go and relax a bit. I also get myself time to take care of my baby and also myself. So I always pray that they are around. In real practice, of course for them they have a fresh mind, and they have read books. Apart from lack of experience, they are better than us since they are still researching. For us who studied sometime back we do not have much theory. So we share knowledge”.* KII, Health Facility Staff, MUST

Additional challenges that were reported to have had an impact on the students’ potential contribution are related to the administrative arrangements for COBERS. Inadequate, and in some cases total absence of transport for outreaches to communities was a key constraint to students’ activities and to reach at some sites. Also, in some cases medicines and other supplies like gloves were limited which meant that students could not effectively meet the demand in the communities.

## Discussion

This study provides evidence that students contribute to health service delivery at health facility and community levels. At facility level, students were perceived to be caring, available and accessible to patients. They also participated in hands-on patient care. Students engaged health workers in intellectual discussions thereby sharing their knowledge and skills. At the community level, students participated in water and sanitation improvement as well as other public health activities including health education and promotion of healthy behaviours including hygiene, sanitation, HIV testing and when to seek treatment. A number of school health activities were also reported. During their COBERS attachments the students certainly get the opportunity to enhance learning by ‘seeing where the patients they see in the health facilities come from in the community’. Thus, students learn how clinical medicine is complemented by public health practice.

**Map 1:**
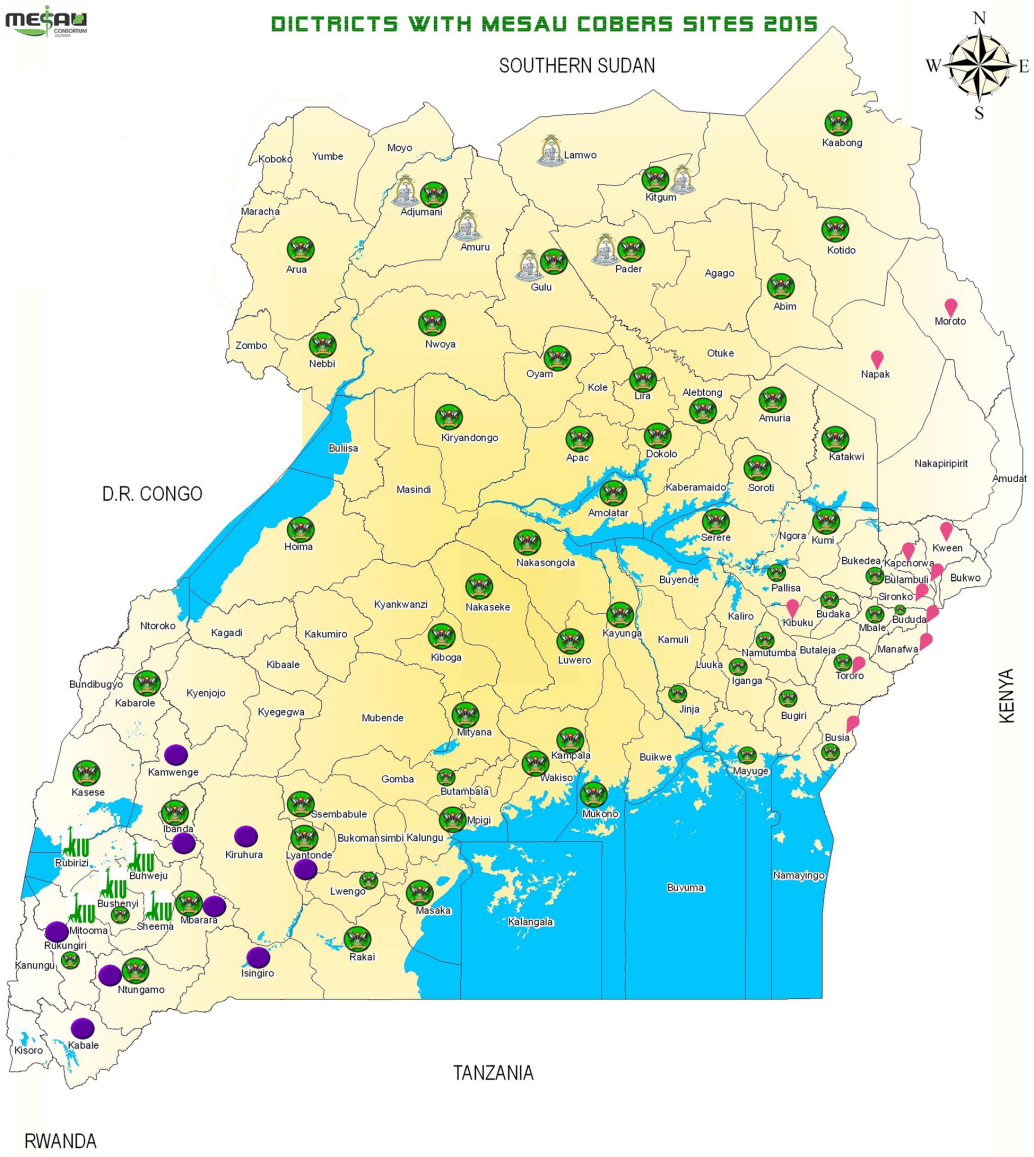
Districts with MESAU COBERS sites, 2015. Districts with Gulu University COBERS sites. Districts with Busitema University COBERS sites. Districts with KIU COBERS sites. Districts with MUST COBERS sites. Districts with MakCHS COBERS sites. *Source: MESAU, MakCHS 2012*

This study highlights the potential of health professions students to contribute to PHC services and to enhance coverage (Map 1). To our knowledge, no attempt has yet been made to document the contribution of pre-service health professions students to service delivery in low income settings. In the USA, undergraduate medical education service-learning has been identified as a unique way of bridging the gap in the provision of specialised health care services such as for children with special care needs [16, 17]. Also, medical student-run clinics (SRCs) were found to be important contributors to health care for disadvantaged patients [18, 19]. Given the gaps in human resources for health (HRH) in Uganda especially in rural areas and at the lower levels of service delivery, and considering the students’ contributions to health service delivery at facilities and in communities we have documented, it is reasonable to conclude that the students, through COBERS are bridging a service delivery gap at PHC level. A clear distinction, however, must be made between the SRCs in the USA and COBERS at the MESAU institutions: firstly, participating in SRCs is voluntary while COBERS is a core, compulsory curriculum component. Secondly, SRCs mostly operate outside the mainstream health service [18] whereas COBERS makes use of health facilities that are part of the mainstream health service. Lastly, student activities during COBERS are supervised while SRCs may or may not be supervised [18].

It is important to note that the services that the students contribute to are mainly at the PHC level. PHC is at the interface between the community and the health system, and is designed to address the major health problems peculiar to a country’s population [20]. Thus, interventions at this level have considerable potential for impact. An examination of the Global Burden of Disease report of 2010 re-emphasises the significance of primary care interventions especially in developing countries where coverage of basic PHC services remains critical [21]. However, shortage of health workers, limited opening hours, long waiting time, poor staff attitude and poor relationships between community and health staff hinder access to these basic health services [22–27]. We have shown that undergraduate health professions students at health facilities contribute to overcoming these barriers. Additionally, community members and facility staff felt that health seeking and utilisation of health services improved as a result. This could be due to the students’ participation in PHC activities including community mobilisation, home and school visits and educational talks which created a demand for facility-based services. Recruitment, training and retention of adequate numbers of human resources to staff PHC centers is critical for achieving universal access to PHC services [28–30]. Ensuring coverage of PHC is fundamental to achieving universal health coverage [31, 32]. Providing the HRH to ensure coverage of PHC therefore becomes critical for achieving universal health coverage [28, 33, 34]. Thus, by contributing to PHC, undergraduate health professions students have the potential to enhance accessibility to and quality of PHC, ultimately contributing to the achievement of universal health coverage.

Although the numbers of health professions students at the MESAU institutions are not large enough to have a sustained impact on health service delivery and coverage of PHC throughout the year, there are at least 63 other health worker training institutions spread all over the country that have community-based education in their curricula [35]. The majority of these are nursing schools. Furthermore, these programs have community outreach activities including immunization, health promotion and community diagnosis as part of community-based education. Many of them follow up community assessments with health promotion or preventative activities to address problems identified [35]. Medical schools in sub-Saharan Africa reported first year enrolment of up to 1800 students per year [36] and in total have 10,000-11,000 graduates per year [37]. These students, together with students of other health worker training programs in sub Saharan Africa present a formidable force if leveraged to contribute to PHC service delivery using the community-based education platform. Moreover, these are long term on-going academic programs and thus present substantial potential for sustainability of student service contributions for generations to come.

While CBE has great potential as a platform for students to contribute to delivery of health services and is therefore desirable, the associated costs can be prohibitive. There are costs attached to accommodation, meals, transport, supervision and providing tutorials. Institutions ought to be aware of the costs and should have strategies for meeting these costs in a sustainable way before rolling-out CBE. Although the MESAU institutions have formal partnerships with the local governments where the participating health facilities are found, these partnerships have so far not involved financial commitments by most local governments whose revenue is also very limited. The linkages with the Ministries of Health and of Education and Sports initiated by MESAU have gained a lot of momentum and interest on both sides. We envisage that the concrete evidence of students’ contribution to service delivery we have documented will provide stronger justification for central and local government support to COBERS. The support could be in terms of accommodation, material supplies and facilitating engagement with stakeholders.

There are a few limitations to consider while interpreting our results. The period of student placement, tutors and supervisors vary across institutions. This could have affected students’ contribution to PHC. Also, even though the tools were standardised and training of research assistants conducted centrally, translations across the different cultural settings could have had some variations. Efforts to back-translate minimised this variability effect.

## Conclusions

This study confirms that health professions students provide positive contributions to PHC services within MESAU institutions. However, students’ contributions have not been previously documented or well-recognized. This study calls for a comprehensive examination of community based education and health service-learning programs in diverse settings in order to further quantify and document the contributions of students, and to delineate the costs and benefits to host institutions and communities. In view of the benefits in terms of student learning outcomes and contributions to service delivery, we propose that all health worker training programs in sub-Saharan Africa embrace community-based education. Other approaches, such as longitudinal rural clinical clerkships may maximise impact and should be explored. There is need for greater government support and investment in order to ensure long-term sustainability of community-based education and its outcomes.

### Availability of data and materials

Data for this article can be accessed from the MakCHS School of Medicine Ethics Review Committee. Contact Ms. Aida Nakawunde: research@chs.mak.ac.ug; aidan.kiseka@gmail.com

## Abbreviations

BU: Busitema University
CBE: Community Based Education
CME: Continuous Medication Education
COBERS: Community-based Education, Research and Service
FGD: Focus Group Discussions
GU: Gulu University
HRH: Human Resources for Health
KII: Key Informant Interview
KIU: Kampala International University
MakCHS: Makerere University College of Health Sciences
MEPI: Medical Education Partnership Initiative
MESAU: Medical Education for Equitable Services to All Ugandans consortium
MUST: Mbarara University of Science and Technology
OPD: Out-patient Department
PHC: Primary Health Care
SRC: Student-run clinics
VHT: Village Health Team

## References

[1] World Health Organisation (1987). Community-based education of health personnel.

[2] Bor D (2003). Position paper on community-based education for health professionals. Educ Health (Abingdon).

[3] Magzoub ME, Schmidt HG (2000). A taxonomy of community-based medical education. Acad Med.

[4] Johnson I, Hunter LM, Chestnutt IG (2012). Undergraduate students' experiences of outreach placements in dental secondary care settings. Eur J Dent Educ.

[5] Ndira S, Ssebadduka D, Niyonzima N, Sewankambo N, Royall J (2014). Tackling malaria, village by village: a report on a concerted information intervention by medical students and the community in Mifumi, Eastern Uganda. Afr Health Sci.

[6] Lalloo R, Evans JL, Johnson NW (2013). Dental students' reflections on clinical placement in a rural and indigenous community in Australia. J Dent Educ.

[7] Hudson JN, Knight PJ, Weston KM (2012). Patient perceptions of innovative longitudinal integrated clerkships based in regional, rural and remote primary care: a qualitative study. BMC Fam Pract.

[8] Kristina TN, Majoor GD, van der Vleuten CP (2006). Comparison of outcomes of a community-based education programme executed with and without active community involvement. Med Educ.

[9] Omotara BA, Padonu MO, Yahya SJ (2004). Assessment of the impact of community-based medical education of the University of Maiduguri on communities in three local government areas of Borno State, Nigeria: community leaders' perspectives. Educ Health (Abingdon).

[10] Omotara BA, Yahya SJ, Shehu U, Bello HS, Bassi AP (2006). Communities' awareness, perception and participation in the Community-Based Medical Education of the University of Maiduguri. Educ Health (Abingdon).

[11] Mbalinda SN, Plover CM, Burnham G, Kaye D, Mwanika A, Oria H, Okullo I, Muhwezi W, Groves S (2011). Assessing community perspectives of the community based education and service model at Makerere University, Uganda: a qualitative evaluation. BMC Int Health Hum Rights.

[12] Kiguli S, Mubuuke R, Baingana R, Kijjambu S, Maling S, Waako P, Obua C, Ovuga E, Kaawa-Mafigiri D, Nshaho J (2014). A consortium approach to competency-based undergraduate medical education in Uganda: process, opportunities and challenges. Educ Health (Abingdon).

[13] Mafigiri DK, Ayebare F, Baingana RK, Okello E, Sewankambo NK (2014). Medical Education for Equitable Services for All Ugandans (MESAU) consortium: development and achievements. Acad Med.

[14] Talib ZM, Baingana RK, Sagay AS, Van Schalkwyk SC, Mehtsun S, Kiguli-Malwadde E (2013). Investing in community-based education to improve the quality, quantity, and retention of physicians in three African countries. Educ Health (Abingdon).

[15] Friese S (2014). Qualitative Data Analysis with ATLAS.ti.

[16] Pakulski LA (2011). Addressing qualified personnel shortages for children who are deaf or hard of hearing with an interdisciplinary service learning program. Am J Audiol.

[17] DeMattei RR, Allen J, Goss B (2012). A service-learning project to eliminate barriers to oral care for children with special health care needs. J School Nurs.

[18] Simpson SA, Long JA (2007). Medical student-run health clinics: important contributors to patient care and medical education. J Gen Intern Med.

[19] Batra P, Chertok JS, Fisher CE, Manseau MW, Manuelli VN, Spears J (2009). The Columbia-Harlem Homeless Medical Partnership: a new model for learning in the service of those in medical need. J Urban Health.

[20] Declaration of Alma-Ata. In: *International Conference on Primary Health Care: 1978; Alma-Ata, USSR*; 1978.

[21] Lim SS, Vos T, Flaxman AD, Danaei G, Shibuya K, Adair-Rohani H, Amann M, Anderson HR, Andrews KG, Aryee M (2012). A comparative risk assessment of burden of disease and injury attributable to 67 risk factors and risk factor clusters in 21 regions, 1990-2010: a systematic analysis for the Global Burden of Disease Study 2010. Lancet.

[22] Mutale W, Bond V, Mwanamwenge MT, Mlewa S, Balabanova D, Spicer N, Ayles H (2013). Systems thinking in practice: the current status of the six WHO building blocks for health system strengthening in three BHOMA intervention districts of Zambia: a baseline qualitative study. BMC Health Serv Res.

[23] Kiguli J, Ekirapa-Kiracho E, Okui O, Mutebi A, Macgregor H, Pariyo GW (2009). Increasing access to quality health care for the poor: Community perceptions on quality care in Uganda. Patient Preference Adherence.

[24] Gourlay A, Birdthistle I, Mburu G, Iorpenda K, Wringe A (2013). Barriers and facilitating factors to the uptake of antiretroviral drugs for prevention of mother-to-child transmission of HIV in sub-Saharan Africa: a systematic review. J Int AIDS Soc.

[25] Thiam S, Kimotho V, Gatonga P (2013). Why are IPTp coverage targets so elusive in sub-Saharan Africa? A systematic review of health system barriers. Malaria J.

[26] Kiwanuka SN, Ekirapa EK, Peterson S, Okui O, Rahman MH, Peters D, Pariyo GW (2008). Access to and utilisation of health services for the poor in Uganda: a systematic review of available evidence. Trans R Soc Trop Med Hyg.

[27] Tafese F, Woldie M, Megerssa B (2013). Quality of family planning services in primary health centers of Jimma Zone, Southwest Ethiopia. Ethiopian J Health Sci.

[28] Nations. U (2012). Report of the United Nations Conference on Sustainable Development. Rio de Janeiro, Brazil, 20–22 June 2012.

[29] Adopting consensus text, General Assembly encourages member states to plan, pursue transition of national health care systems towards universal coverage. GA/11326, 2012 [http://www.un.org/News/Press/docs/2012/ga11326.doc.htm]. Accessed 15 Feb 2015.

[30] Health in the Post-2015 Development Agenda. Report of the Global Thematic Consultation on Health. [http://www.worldwewant2015.org/health]. Accessed 15 Feb 2015.

[31] Berman R, Powe C, Carnevale J, Chao A, Knudsen J, Nguyen A, Edgman-Levitan S (2012). The crimson care collaborative: a student-faculty initiative to increase medical students' early exposure to primary care. Acad Med.

[32] Shelton JD (2013). Ensuring health in universal health coverage. Nature.

[33] Miller C, Holly L (2012). Health workers and universal health coverage. Lancet.

[34] Alliance. GHW (2013). Universal Health Coverage: The ‘Grand Challenge’ of Human Resources for Health. Key messages presented to the WHO/ World Bank Ministerial-level Meeting on Universal Health Coverage, Geneva, 18-19 February 2013.

[35] Kaye DK, Muhwezi WW, Kasozi AN, Kijjambu S, Mbalinda SN, Okullo I, Nabirye RC, Oria H, Atuyambe L, Groves S (2011). Lessons learnt from comprehensive evaluation of community-based education in Uganda: a proposal for an ideal model community-based education for health professional training institutions. BMC Med Educ.

[36] Chen C, Buch E, Wassermann T, Frehywot S, Mullan F, Omaswa F, Greysen SR, Kolars JC, Dovlo D, El Gali Abu Bakr DE (2012). A survey of Sub-Saharan African medical schools. Hum Resourc Health.

[37] Mullan F, Frehywot S, Omaswa F, Sewankambo N, Talib Z, Chen C, Kiarie J, Kiguli-Malwadde E (2012). The Medical Education Partnership Initiative: PEPFAR's effort to boost health worker education to strengthen health systems. Health Aff (Millwood).

